# Delivery of cell-penetrating peptide-peptide nucleic acid conjugates by assembly on an oligonucleotide scaffold

**DOI:** 10.1038/srep17640

**Published:** 2015-11-27

**Authors:** Xing-Liang Zhao, Bi-Cheng Chen, Jin-Chao Han, Lai Wei, Xiao-Ben Pan

**Affiliations:** 1 Peking University People’s Hospital, Peking University Hepatology Institute, Beijing Key Laboratory of Hepatitis C and Immunotherapy for Liver Diseases; Beijing 100044, P.R. China; 2 Zhejiang Provincial Top Key Discipline in Surgery, Wenzhou Key Laboratory of Surgery; Department of Surgery, The First Affiliated Hospital, Wenzhou Medical University, Wenzhou, 325200, P.R. China

## Abstract

Delivery to intracellular target sites is still one of the main obstacles in the development of peptide nucleic acids (PNAs) as antisense-antigene therapeutics. Here, we designed a self-assembled oligonucleotide scaffold that included a central complementary region for self-assembly and lateral regions complementing the PNAs. Assembly of cell-penetrating peptide (CPP)-PNAs on the scaffold significantly promoted endocytosis of PNAs by at least 10-fold in cell cultures, particularly for scaffolds in which the central complementary region was assembled by poly(guanine) and poly(cytosine). The antisense activity of CPP-PNAs increased by assembly on the scaffold and was further enhanced after co-assembly with endosomolytic peptide (EP)-PNA. This synergistic effect was also observed following the assembly of antigene CPP-PNAs\EP-PNAs on the scaffold. However, antigene activity was only observed by targeting episomal viral DNA or transfected plasmids, but not the chromosome in the cell cultures. In conclusion, assembly on oligonucleotide scaffolds significantly enhanced the antisense-antigene activity of PNAs by promoting endocytosis and endosomal escape. This oligonucleotide scaffold provided a simple strategy for assembly of multiple functional peptide-PNA conjugates, expanding the applications of PNAs and demonstrating the potential of PNAs as antiviral therapeutics.

Peptide nucleic acids (PNAs) are a class of DNA mimics having a pseudopeptide backbone. Despite dramatic differences in the chemical composition of the backbone, PNA forms Watson-Crick bonds with DNA and RNA with higher thermal stability than natural duplexes due to the lack of electrostatic repulsion. Furthermore, PNAs are extremely stable because they are able to resist to degradation by proteases and nucleases^1^,^2^. These qualities make PNA molecules promising candidates for clinical applications as regulators of gene expression^3^,^4^.

The ability of antisense PNAs (asPNAs) to bind to target RNA has already been demonstrated, and asPNAs have been shown to potently and selectively inhibit gene expression in cells and animals^5^,^6^,^7^,^8^,^9^,^10^. PNAs designed to target the DNA coding strand also show antigene capacity, which is remarkably efficient in cell-free systems; however, success is less certain in complex cellular environments^3^,^11^. Transcriptional start sites are one potential target of PNAs, in which the open complex formed by the RNA polymerase is likely to create a single-stranded region that is susceptible to binding by PNAs. Polypyrimidine-polypurine sequences may also be targeted by bisPNAs through strand invasion and formation of a four-stranded complex. Furthermore, supercoiled DNA can be hybridized by PNAs containing mixtures of A, C, T, and G. Hybridization is promoted by the negative torsional stress of supercoiling and is most efficient within AT-rich regions and at inverted repeats capable of forming cruciforms^12^,^13^,^14^,^15^,^16^,^17^,^18^,^19^. This invasion of duplex DNA by the neutral PNA backbone suggests that antigene PNAs (agPNAs) may be important agents for inhibiting the transcription of genes within cells.

However, exploration of the potential of PNAs as drugs in gene therapy has been hampered by the poor intrinsic uptake of PNA by living cells. As a large hydrophilic molecule, PNA does not cross lipid membranes easily. A variety of cellular delivery systems have been developed during the last few years. These include microinjection, electroporation, cotransfection with DNA, or conjugation to lipophilic moieties, nanoparticles, cell-penetrating peptides (CPPs), oligo-aspartic acid, or nuclear localization signal (NLS) peptides to enhance cellular internalization. Because cell membranes have a negative charge, cationic transfection reagents need to be used to cotransfect the PNA/DNA complex^20^,^21^,^22^,^23^,^24^,^25^. Furthermore, delivery into the cytosolic space and nucleus remains challenging. A high concentration of CPP-PNA conjugates is needed to initiate endocytosis, but the conjugates often remain trapped inside the endosomes. While adding calcium ions, chloroquine (CQ), or sucrose facilitates the release of PNAs from endosomes in cell culture, these strategies are not clinically applicable^26^,^27^,^28^,^29^,^30^.

In this study, in order to overcome the cell membrane barrier and endosomal entrapment of intracellular CPP-PNAs, we designed a self-assembled oligonucleotide scaffold that was capable of assembling specifically with multiple PNA conjugates modified by various functional moieties. We used the hepatitis B virus (HBV) genome as a target for evaluating the activity of the PNA-oligonucleotide scaffold complex in various cell lines.

## Results

### Experimental design

**Figure 1:**
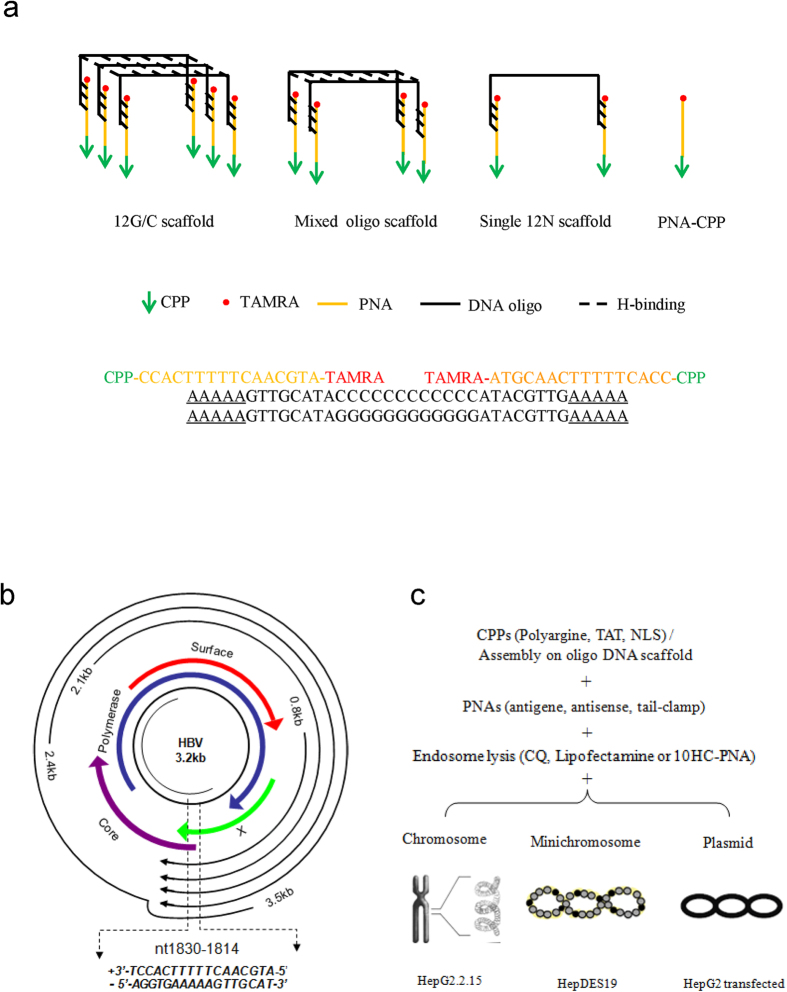
Design of the oligo DNA scaffold and target genes of antisense or antigene PNAs. (**a**) A schematic of the carrier oligo DNA scaffold. The oligonucleotide scaffold has a central self-complementary area for assembly with PNAs and two flanks, which are complementary to the target PNA. The lower plot shows the sequences of oligo DNA (12G/C) and PNA. (**b**) Nucleotides 1814–1830 of HBV DNA served as the target sequence for antigene or antisense PNAs. (**c**) The target HBV DNA was present in different forms, including episomal viral covalently closed circular DNA (cccDNA) in HepDES19 cells, the pUC18-HBV1.2 plasmid transfected into HepG2 cells, and chromosomes in HepG2.2.15 cells integrated with HBV DNA.

To increase the local concentration of CPPs, we designed a carrier oligonucleotide scaffold to recruit multiple CPP-PNAs (Fig. 1a). The carrier oligo had a central complementary area that could form a duplex or triplex strand with other oligo DNA and two flanks that were complementary to the PNAs. The sequences of the central region included 12 As, Ts, Cs, Gs, or mixed-sequenced oligonucleotides. Different lengths of flanking oligonucleotides were tested to identify the appropriate balance of the association and disassociation between the PNAs and oligonucleotides; this was expected to affect the assembly of the PNA-oligonucleotide scaffold in the test tube and disassociation of the PNAs from the oligo scaffold following endocytosis. Oligos with random sequences at the flanking regions were designed as control carriers (Fig. 1a, Table 1).

Upon infection of hepatocytes, HBV DNA is transported to the nucleus, where it is converted to a supercoiled covalently closed circular DNA (cccDNA). The episomal cccDNA is a storage pool of the viral genome, serving as the template for transcription of the pregenomic RNA and the three main subgenomic RNAs^31^. In the present study, the target HBV DNA was introduced into the cells in different forms, including cccDNA in HepDES19 cells^32^, the pUC18-HBV1.2 plasmid transfected into HepG2 cells, and chromosomal DNA in HepG2.2.15 cells integrated with HBV DNA. Nucleotides 1814–1830 of HBV DNA served as the targeting sequence for antigene or antisense PNAs (Fig. 1b,c). This region included the core promoter/enhancer I area of the HBV genome, the transcription start site of the HBV e antigen (HBeAg), an exocrine protein that can be readily detected in supernatants; and a polypyrimidine-polypurine area that could serve as a target of PNA-clamping for strand invasion and formation of a four-stranded complex^33^. A mismatched PNA containing a two-base substitution was designed as control PNA.

### Characterization of the assembled oligonucleotide scaffolds

**Figure 2:**
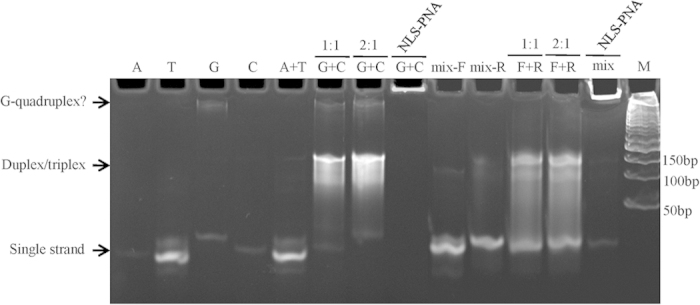
Assessment of assembly of oligonucleotide scaffolds and binding to the complementary PNA. Single-strand oligomers (lanes 1–4), mixed sequences (lanes 9 and 10), the oligomer 12A-5A plus 12T-5A (lane A + T), the oligomer 12G-5A plus 12C-5A (lane G + C, ratio of 1:1 or 2:1), or mixed-sequences forward plus reverse (lane F + R, ratio of 1:1 or 2:1) were annealed in a total volume of 20 μL and analyzed by polyacrylamide gel electrophoresis. Lanes 8 and 13 represent the equimolar complementary NLS-PNA (final concentration of 2.5 μM) annealed with the oligonucleotide scaffold. The bands corresponding to single strand, double/triplex strand, or G-quadruplex are indicated (arrows).

In order to validate the assembly feature of these oligos and the target binding efficiency of PNA to the scaffolds, the oligos were annealed with or without NLS-PNAs and analyzed using polyacrylamide gel electrophoresis. Because positively charged CPP-PNA binds DNA more strongly at low salt concentrations, the annealing was performed in a low ionic strength buffer and gradually cooled down to 35 °C^34^. As shown in Fig. 2, for the single stranded oligos, apparent bands were only observed for oligo 12T-5A; this may have resulted from the self-pairing of the central 12T with flanking 5A. Interestingly, a weak but large band was observed in all lanes containing 12G-5A, which may indicate the formation of a G-quadruplex because of the 12 Gs in the central area. In the annealed 12G-5A/12C-5A or mixed-sequence oligo, a smear band was observed around 100–150 bp, which was much larger than the length of the oligo (approximately 40 nucleotides). In these annealed oligos, the double-stranded form was only formed in the central area, and the flanks remained free. This configuration delay migration during electrophoresis. As the ratio of 12G-5A to 12C-5A increased to 2:1, the intensity of the annealed band apparently increased. However, no changes were observed in the mixed-sequence oligos, which may indicate the presence of the triplex-stranded form in the annealed 12G-5A/12C-5A but not in the mixed-sequence oligos. While NLS-PNA was annealed with these oligos, the DNA bands were completely retained in the sample well, demonstrating the high efficiency of the binding interaction between NLS-PNAs and oligonucleotide scaffolds.

### Assembly of NLS-PNAs on oligonucleotide scaffold promoted endocytosis

**Figure 3:**
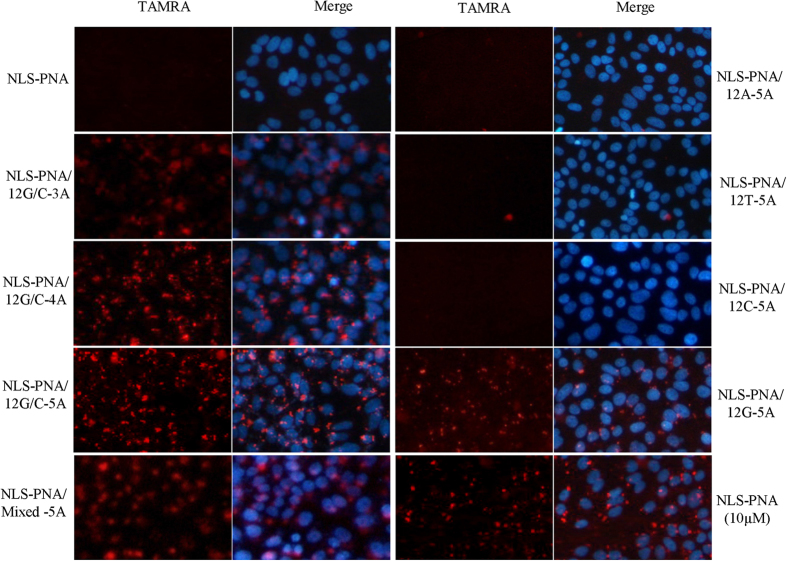
Assembly of NLS-asPNAs on the oligonucleotide scaffold markedly improved internalization in cell cultures. HepG2 cells at 50–60% confluence were treated with the PNA-CPPs/oligo scaffold complex by incubation in OPTI-MEM containing the PNAs for 4 h. Cells were further incubated for 20 h after supplementation with the same volume of growth medium containing 10% FBS. DAPI was used for nuclear staining. The concentration of NLS-PNAs used for assembly with the oligonucleotide scaffold was 1 μM, except when NLS-PNAs were used alone (1 or 10 μM).

To determine whether the entry of PNA-CPPs into cells was improved by assembly with oligonucleotide scaffolds, the penetration properties of NLS-PNAs labeled with TAMRA were assessed in HepG2 cells at 24 h after treatment using fluorescence microscopy (Fig. 3). In HepG2 cell treated with NLS-PNAs alone, no intracellular fluorescence was detected at 1 μM of NLS-PNAs, and such fluorescence was only detected after exposure to a high concentration (10 μM) of NLS-PNAs. The signal was significantly enhanced when NLS-PNAs were assembled on the oligonucleotide scaffold. When used at a concentration of 1 μM NLS-PNAs, the strongest signal was detected in cells treated with NLS-PNAs assembled on the oligonucleotide scaffold 12G/C-5A (2:1), consistent with the results of electrophoresis of oligos. Weaker signals were detected when NLS-PNAs were assembled on the mixed-sequence oligo DNA, which theoretically formed double-stranded DNA and assembled with four NLS-PNA molecules. No signal was detected when the NLS-PNAs were assembled on the 12T/A or on single-stranded oligos including a central 12A, 12T, or 12C. However, cell entry was detected when NLS-PNAs was assembled on the single-stranded oligonucleotide with a central 12G, suggesting a clustering of NLS-PNAs on the G-quadruplex. When the length of the complementary flanking regions was adjusted by the number of adenosines at the terminal region, longer regions of complementary base pairs increased the efficiency of PNA intracellular delivery. However, all of these PNA-peptide conjugates exhibited a punctate distribution in the cytoplasm, indicating that they remained localized in the endosomes.

### Co-assembly of NLS-PNAs and EP-PNAs enhanced antisense activity

**Figure 4:**
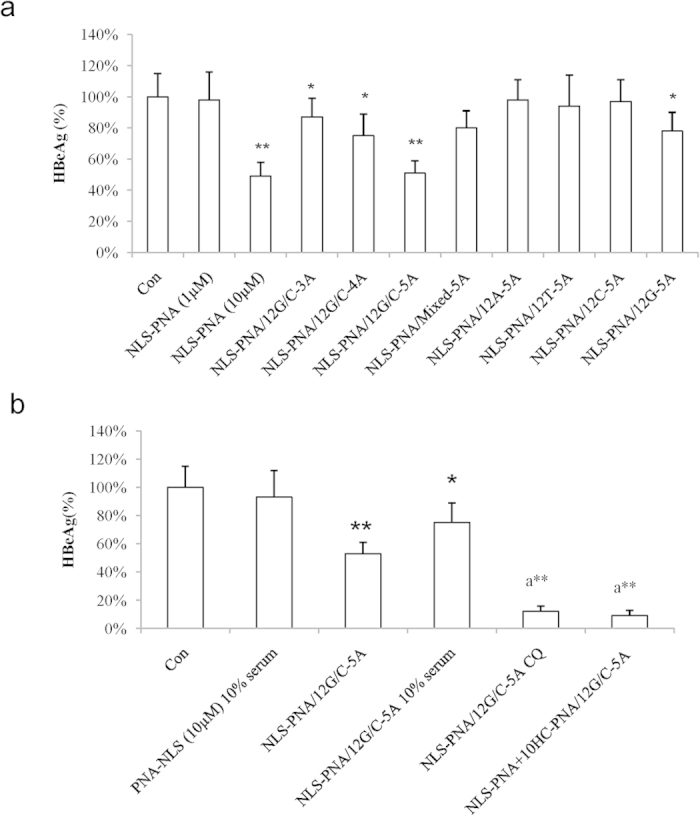
Assembly of NLS-PNAs on the oligonucleotide scaffold enhanced antisense activity. (**a**) The antisense activities of 1 μM NLS-asPNAs assembled on different oligonucleotide scaffolds and of 1 or 10 μM NLS-PNAs without assembly were evaluated in the HBV DNA-integrated cell line HepG2.2.15 for 3 days. (**b**) HepG2.2.15 cells were treated with NLS-PNAs assembled on the scaffold 12G/C-5A, and the conditions of incubation are indicated on the x-axis. HBeAg in the supernatant was detected for evaluation of antisense activity (n = 3). Comparisons between two groups were carried out with Student’s t-tests. *p < 0.05, **p < 0.01 compared with Con, a**p < 0.01 compared with NLS-PNA/12G/C-5A.

To evaluate whether the activity of antisense CPP-PNAs was improved by the assembly on oligonucleotide scaffolds, we tested the antisense activity of the NLS-PNAs in HepG2.2.15 cells, which harbored the 1.3-fold full-length HBV genome integrated within the native genome^35^. Antisense effects were consistent with the strength of the intracellular fluorescent signal. As shown in Fig. 4, HBeAg in culture medium was inhibited by approximately 50% in cells treated with 10 μM NLS-asPNA or 1 μM NLS-asPNA assembled on the 12G/C-5A. HBeAg was inhibited by 15–25% in cells treated with 1 μM NLS-PNAs assembled on 12G/C-3A, 12G/C-4A, or single-stranded 12G-5A (Fig. 3a).

HBeAg was inhibited by 90% following co-incubation with NLS-PNAs assembled on the 12G/C-5A oligo scaffold in the presence of the endosomolytic agent CQ. Similar effects were observed when NLS-PNAs were co-assembled on the scaffold with 10HC-PNA, which included histidine-rich endosomolytic peptide^36^. Furthermore, no inhibition of HBeAg was detected in HepG2.2.15 cells treated with 10 μM NLS-PNAs in medium containing 10% fetal bovine serum (FBS). However, HBeAg was inhibited by 20% in HepG2.2.15 cells treated with 1 μM NLS-PNA assembled on the 12G/C-5A oligo in medium containing 10% FBS (Fig. 3b).

### CPP-agPNAs exhibited antigene activity to episomal viral DNA

**Figure 5:**
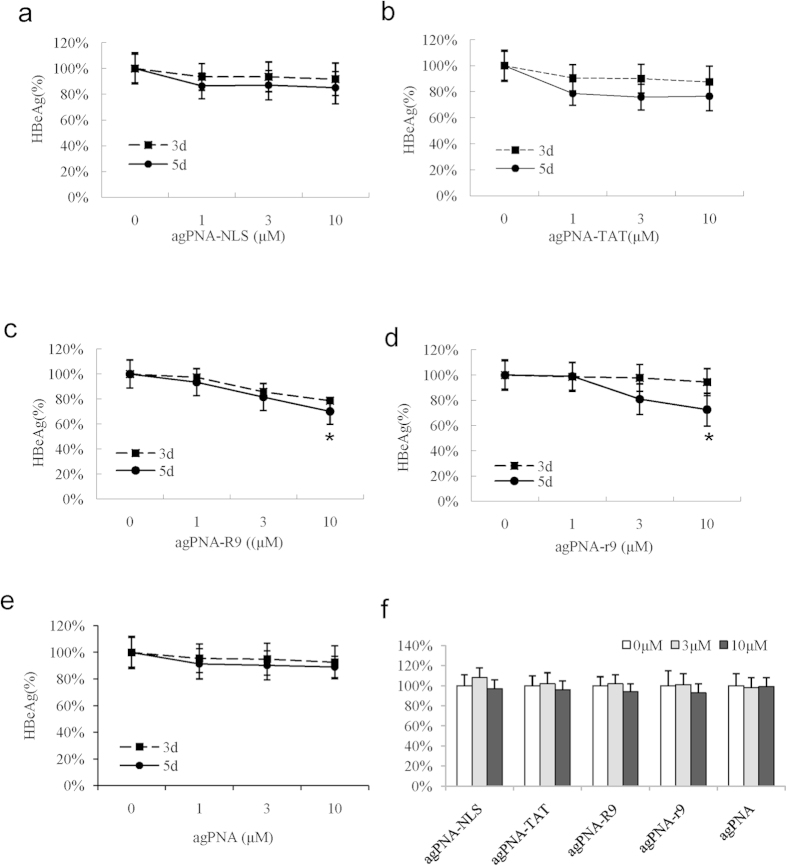
Antigene activity of CPP-agPNAs in HepG2.2.15 cells. The antigene activity of agPNAs conjugated with different CPPs was tested in HepG2.2.15 cells. The cells were treated for 5 days, and medium was completely changed on day 3. HBeAg in the supernatant was detected for evaluation of antigene activity (n = 3) on days 3 and 5. Cell viability was determined after CPP-PNA treatment using MTT assays (**f**).

Next, we tested the antigene activity of CPP-agPNAs in HepG2.2.15 cells. Although NLS-asPNAs exhibited substantial antisense activity in HepG2.215 cells, no inhibition of HBeAg was detected in cells treated with NLS-agPNAs, even at a high concentration (10 μM). When agPNAs were conjugated with several other CPPs, including TAT, R9, and r9, HBeAg was very weakly inhibited, if at all, in cells treated with 10 μM R9-agPNA or r9-agPNA on day 5 (Fig. 5).

**Figure 6:**
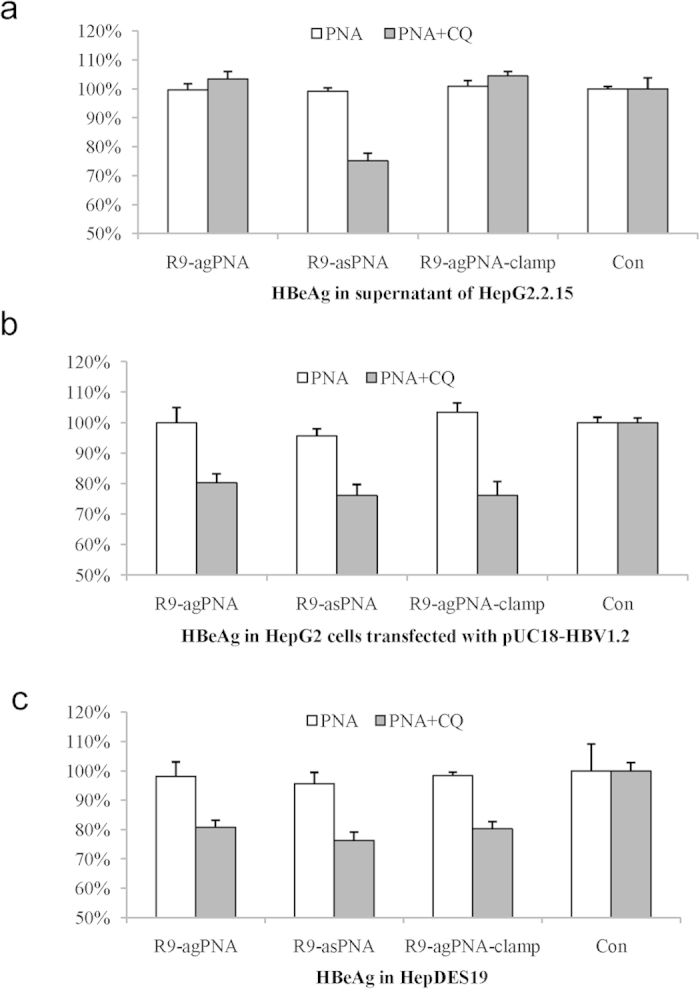
Antigene activity of R9-PNAs targeting HBV DNA in different cell lines for 3 days. HepG2.2.15 cells integrated with HBV DNA in the chromosome (**a**), HepG2 cells transfected with the pUC18-HBV1.2 plasmid (**b**), and HepDES19 cells containing episomal HBV cccDNA (**c**) were treated with 1 μM antigene or antisense R9-PNAs with or without chloroquine (CQ).

The inhibition of HBeAg by CPP-asPNA but not CPP-agPNA in the HepG2.2.15 cells indicated that asPNAs may specifically block the translation of mRNA. However, agPNAs could not invade into the mixed double-stranded DNA in the chromosome. Mixed-sequence PNAs have been shown to be capable of invading supercoiled plasmid DNA and certain regions of chromosomal DNA. Whether the supercoiled minichromosome HBV cccDNA is prone to invasion by agPNAs as supercoiled plasmids remains to be elucidated. Thus, we further tested these CPP-agPNAs in HepG2 cells transfected with the pUC18-HBV1.2 plasmid and in HepDES19 cells in which HBeAg was only produced from episomal HBV cccDNA in the nucleus (Fig. 6). HBeAg in the supernatant was inhibited by approximately 25% in all three cell lines treated with 1 μM asPNAs with added CQ. However, inhibition of HBeAg by agPNAs was detected in HepDES19 and HepG2 cells transfected with pUC18-HBV1.2, but not in HepG2.2.15 cells. Exposure to agPNAs with tail clamping did not enhance antigene activity.

### Assembly of agPNAs on the oligonucleotide scaffold enhanced antigene activity

**Figure 7:**
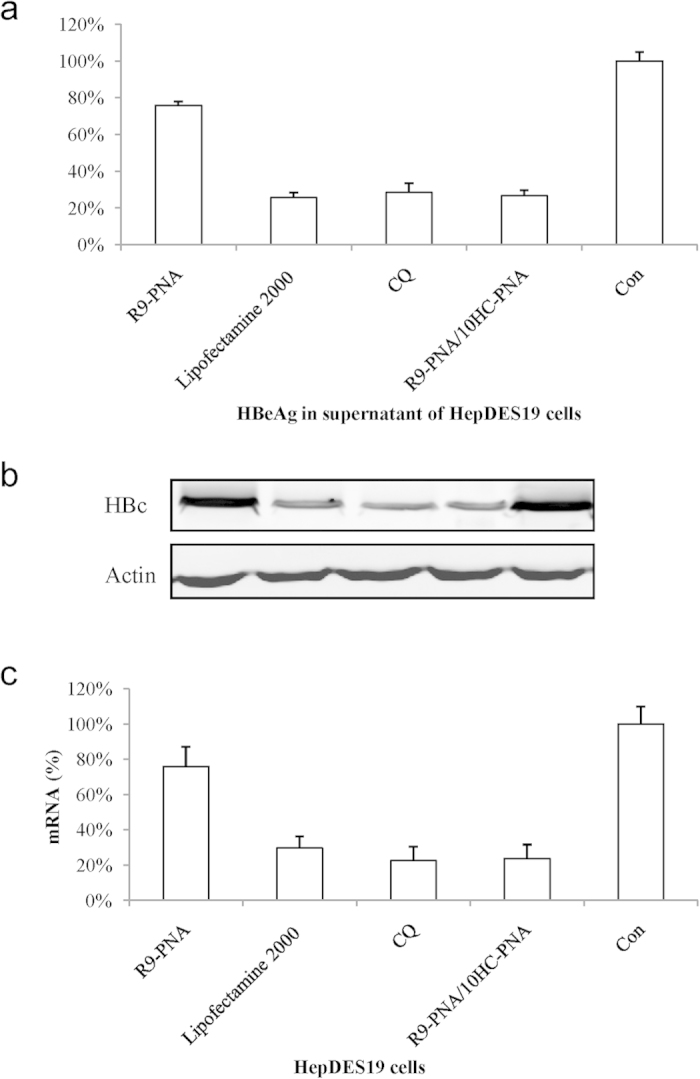
Assembly of antigene R9-PNAs on the oligonucleotide scaffold enhanced the antigene activities of HepDES19 cells. HepDES19 cells were transfected with 1 μM R9-PNAs assembled on the oligo DNA scaffold using Lipofectamine 2000 reagent, CQ, or co-assembly with 10HC-agPNAs. HBeAg in the supernatant (**a**) and intracellular HBV core protein (HBc) (**b**) and HBV mRNA (**c**) were analyzed for evaluation of antigene activity.

To test whether assembly of CPP-agPNAs on the 12G/C-5A oligo scaffold improved antigene activity, R9-PNAs assembled on the oligo DNA scaffold were transfected into HepDES19 cells with Lipofectamine 2000 or CQ or by co-assembly with 10HC-agPNAs. While HBeAg was inhibited by 20% in HepDES19 cells treated with 1 μM agPNAs, addition of CQ, transfection with Lipofectamine 2000, or pre-assembly of R9-agPNAs on the 12G/C-5A oligo scaffold inhibited HBeAg expression by 80% (Fig. 7a). These effects were also confirmed by western blotting for intracellular HBV core protein expression (Fig. 7b). Similar inhibition of viral mRNA was detected using of a quantitative real-time PCR (Fig. 7c).

## Discussion

Penetration of the cell membrane and escape from endosomal entrapment are the main challenges faced in the delivery of PNAs into intracellular target sites. In the present study, we developed a self-assembled oligonucleotide scaffold that was capable of assembling with multiple peptide-PNAs and greatly improved the efficiency of CPP-PNA uptake and endosomal escape.

Firstly, assembly of CPP-PNAs on the oligo scaffold greatly increased the efficiency of cellular endocytosis. Endocytosis of CPP cargo can be triggered by electrostatic interactions with cell surface glycosaminoglycans (GAGs). When bound to GAGs, cargo may enter following the recycling cycle of GAGs or enter more effectively via clustering of GAGs, which activate intracellular signals and actin remodeling^37^,^38^,^39^,^40^. This suggests that CPP-PNAs clustered on the scaffold activate the clustering of GAGs, which may explain the enhanced endocytosis. Compared with the other formulations, PNAs assembled on the scaffold by central poly(G) and central poly(C) exhibited the strongest signal. A duplex formed between the 12G and 12C strands would have a much higher melting temperature compared with that of the mixed sequence duplex or 12A:12T duplex; this may explain the results observed for cell penetration. Furthermore, 12G/C-5A at ratio of 2:1 produced a much stronger band than that at ratio of 1:1, indicating that oligomers of homoguanine and homocytosine may form triple helices based on Watson-Crick base pairs and by binding in the major groove through Hoogsteen base pairing. This triplex would be expected to cluster with six CPP-PNA molecules and may also be involved in the enhanced endocytosis. Interestingly, single strands of poly(G) with two flanks, but not strands of T, A, or C, resulted in significantly improved efficiency of CPP. Four guanine bases can associate through Hoogsteen hydrogen bonding to form a square planar structure called a guanine tetrad, and two or more guanine tetrads can stack on top of each other to form a G-quadruplex^41^. The results of electrophoresis suggested that a small amount of oligo 12G may form G-quadruplexes, which could cluster multiple CPP-PNAs and promote endocytosis. The melting temperature of the oligo strand of 12T/A was approximately 24 °C, which may explain the lack of effect as the scaffold would be expected to be dissociated following incubation at 37 °C. Moreover, the presence of serum in medium can significantly inhibit the uptake of CPP-PNAs^29^. However, the oligonucleotide scaffold still significantly improved CPP-PNA delivery in the presence of serum, indicating that the negative charge of the oligonucleotide may neutralize the positive charge of CPP and reduce nonspecific ionic and hydrophobic interactions. In addition, the efficiency of endocytosis was significantly regulated by the complementary length of PNA and DNA, indicating that very careful optimization of the complementary region to DNA oligos is necessary for each individual CPP-PNA.

Secondly, the oligo scaffold was easy to assemble with PNAs modified by different functional peptides. PNA-CPPs displayed a punctate distribution inside cells, suggesting an endosomal entrapment. Although CQ and PEI facilitate the escape of endocytosed cargo to the cytosol *in vitro*, these reagents are not a feasible method for *in vivo* alterations. Several peptides have been reported to exhibit endosomolytic properties and have been used for oligonucleotide delivery. Among them, histidine has been used to translate the proton sponge activity of PEIs to peptide-based gene delivery vectors^36^. The co-assembly of 10HC-PNAs and CPP-PNAs resulted in significant improvement of antisense activity, even at low nanomolar concentrations, suggesting that the protonation of histidines generated acidification, thereby causing membrane perturbation and leading to eventual leakage of the endosomal contents.

Finally, our data showed that agPNAs exhibited marked antigene activity against genome-specific targets in the episomal viral DNA, and conjugation with the oligonucleotide scaffold also significantly improved this activity. Nuclear HBV cccDNA represents a major barrier to viral eradication in patients with chronic hepatitis B receiving currently available antiviral therapeutics. Although antisense strategies, including oligonucleotides and small-interfering RNA (siRNA)-mediated gene silencing, have been used to degrade HBV RNAs and eliminate viral proteins, these strategies are not able to remove the stable nuclear cccDNA^42^. The interference of the cccDNA template may provide a more efficient method for inhibition of gene expression than targeting at numerous mRNAs. Our results demonstrated recognition of mixed sequences in cccDNA by antigene PNAs inside cells, suggesting that viral DNA presents an accessible target for antigene strategies.

In conclusion, this oligonucleotide scaffold greatly improved the efficiency of PNA delivery by assembling multiple functional peptides. A remarkable advantage is that the transfection and endosomolytic reagents, which are generally highly cytotoxic, are not necessary for delivery this oligo scaffold/peptide-PNAs complex. This expands the applications of PNAs and indicates the therapeutic potential of antigene PNAs as antiviral reagents to inhibit transcription from episomal viral DNA.

## Materials and Methods

### Preparation of CPP-PNAs and oligonucleotides

All CPP-PNA conjugates (purity >95%) were prepared using a chemical synthesis method (Panagene Co., Daejeon, South Korea). CPP-PNA powder was dissolved in pure water at a concentration of 200 μM as a stock solution and frozen at –20 °C. The sequences of PNAs, CPPs, and oligonucleotides (SBSbio Co., Beijing, China) are listed in Table 1. CPPs were covalently linked to the NH_2_ terminus of PNAs with an O linker. TAMRA-labeled CPP-PNAs were synthesized by substitution of a TAMRA fluorophore to the C terminus in order to determine the transfer and location of complexes.

To assemble the CPP-PNAs with oligonucleotides, CPP-PNAs at a final concentration of 50 μM and oligonucleotides at having equimolar concentrations of complementary strands were annealed in buffer (10 mM Tris, 10 mM NaCl, 1 mM EDTA, pH 7.0). The 12G:12C ratio to form a triplex strand was 2:1, whereas that for 12T:12A and the mixed-sequence was 1:1. Hybridization was performed in a thermal cycler with the following temperature reduction profile: 95 °C, 5 min; 85 °C, 1 min; 75 °C, 1 min; 65 °C, 5 min; 55 °C, 1 min; 45 °C, 1 min; 35 °C, 5 min. To validate the assembly efficiency of PNA-oligonucleotide scaffold, NLS-PNAs co-assembled with oligonucleotide scaffolds were analyzed by gel electrophoresis on a 20% polyacrylamide gel. The gel was stained with a 1% ethidium bromide solution and visualized on a Bio-Rad Gel Doc XR (Hercules, CA, USA).

### Cell cultures and CPP-PNA treatment

Human hepatoma HepG2 cells were maintained in DMEM (Invitrogen, Carlsbad, CA, USA) supplemented with 10% FBS, 100 U/mL penicillin, and 100 μg/mL streptomycin. HepDES19 cells, in which HBeAg was expressed from enriched cccDNA but not from integration of the HBV genome into chromosomes, were kindly provided by Ju-Tao Guo (Drexel University, Philadelphia, PA, USA). HepG2.2.15 and HepDES19 cells were cultured as described above for HepG2 cells except for the addition of 380 μg/mL G418 (Sigma, St. Louis, MO, USA) to the medium. Where needed, tetracycline was routinely added at 1 μg/mL in HepDES19 cells to suppress HBV pgRNA transcription from integrated HBV DNA.

Cells at 50–60% confluence were treated with the PNA-CPP/DNA complex by incubation in OPTI-MEM (Invitrogen) containing PNAs for 4 h, following by an additional 20-h incubation after supplementation with the same volume of growth medium containing 10% FBS. For endosome disruption treatment, 100 μM CQ (Sigma) was added to the OPTI-MEM medium. For transfection, the CPP-PNA/DNA complex was formed using Lipofectamine 2000 *in vitro* reagent (Invitrogen).

### Detection of cellular uptake of CPP-PNAs by fluorescence microscopy

The intracellular distribution of PNAs in HepG2 cells was determined using TAMRA-labeled CPP-PNAs. HepG2 cells were seeded in 8-well chamber slides and incubated with CPP-PNA/DNA complexes overnight. Cells were fixed in 0.4% paraformaldehyde, and cell membranes were permeabilized using 0.2% Triton-X 100 in PBS. 4′,6-Diamino-2-phenylindole dihydrochloride (DAPI; Sigma) was used for staining nuclei. Images of cells were captured using a fluorescence microscope (AX80TF; Olympus, Tokyo, Japan).

### Detection of HBV proteins and HBV RNA

HBV viral proteins and RNAs were detected to assess the antiviral activities of these CPP-PNA/oligo scaffold complexes. HBeAg in the culture medium was detected using an electrochemical illuminescent immunoassay kit on an ARCHITECT i2000 Automatic Immunoassay Analyzer (Abbott Labs, IL, USA). Intracellular HBV core protein was analyzed using western blotting. Cell lysates were separated on 12% sodium dodecyl sulfate-polyacrylamide gels and transferred to polyvinylidene difluoride membranes. Membranes were probed with antibodies against HBc (1:3000; Abcam, Cambridge, UK) and β-actin (1:5000; Abcam). HBV RNA was converted into cDNA and detected using qRT-PCR (Faststar SYBR Master kit; Roche, Mannheim, Germany). β-Actin was used as a housekeeping gene for normalization in qRT-PCR and western blotting. The primers for detection of actin were 5′-GCGCGAAATCGTGCGTGACATT-3′ (forward) and 5′-GATGGAGTTGAAGGTAGTTTCGTG-3′ (reverse), and primers for detection of HBV were 5′-GCTTTGGGGCATGGACATT-3′ (forward) and 5′-CATGCCGTCACCCCAACAC-3′ (reverse).

### Cell viability

Cell viability was determined using a standard 3-(4,5-dimethylthiazol-2-yl)- 2,5-diphenyltetrazolium bromide (MTT)-based Cell Titer 96 Non-Radioactive Cell Proliferation Assay (Promega, Madison, WI, USA) according to the manufacturer’s instructions.

### Statistical analysis

Comparisons between two groups were carried out with Student’s t-tests, and comparisons between multiple groups were analyzed by one-way analysis of variance. Differences with *P* values of less than 0.05 were considered statistically significant.

## Additional Information

**How to cite this article**: Zhao, X.-L. *et al*. Delivery of cell-penetrating peptide-peptide nucleic acid conjugates by assembly on an oligonucleotide scaffold. *Sci. Rep*. **5**, 17640; doi: 10.1038/srep17640 (2015).

## Figures and Tables

**Table 1:** Sequence of CPP-PNAs and DNA oligos.

Type	Name	Sequence
PNAs	Antigene	ATGCAACTTTTTCACC
	Tail-clamp	CACCATGCAACTTTTTC-AEEA-CTTTTTC
	Antigene control	ATGC*T*ACTT*A*TTCACCT
	Antisense	AGGTGAAAAAGTTGCAT
	Antisense control	AGGTGAA*T*AAGT*A*GCAT
Peptides	NLS	PKKKRKV-CONH2
	TAT	RKKRRQRRRPP-CONH2
	Arg(9)	RRRRRRRRR-CONH2
	d-Arg(9)	rrrrrrrrr-CONH2
	10HC	HHHHHHHHHHC
DNA	agDNA scaffold	A*A*AAAGTTGCATA-(12N)-ATACGTTGAAAA*A*
	asDNA scaffold	T*T*TTTCAACGTAT-(12N)-TATGCAACTTTT*T*
	agMixed-F	AAAAAGTTGCATA-CGTTCGTTCGTTCG-ATACGTTGAAAAA
	agMixed-R	AAAAAGTTGCATA-CGAACGAACGAACG-ATACGTTGAAAAA

Note: N represents the oligo A, T, C, or G. *indicates the number of A or T varied from 3 to 5.
